# 662. Using Machine Learning to Aid in the Diagnosis of Multisystem Inflammatory Syndrome in Children

**DOI:** 10.1093/ofid/ofab466.859

**Published:** 2021-12-04

**Authors:** Maulin Soneji, John Tan, Emily Wong

**Affiliations:** Loma Linda University, Loma Linda, California

## Abstract

**Background:**

Multisystem inflammatory syndrome in children (MIS-C) is a newly recognized inflammatory syndrome that occurs post Severe Acute Respiratory Syndrome-Coronavirus-2 (SARS-CoV-2) infection. It affects multiple organ systems - particularly cardiac, gastrointestinal, dermatologic and neurologic. Clinicians may have difficulty diagnosing MIS-C due to its novelty and similarity to Kawasaki disease. Our goal was to use machine learning to predict whether children would have MIS-C based on symptoms and laboratory values.

**Methods:**

A retrospective review was conducted of patients admitted to Loma Linda University Children’s Hospital who were suspected of having MIS-C. Demographic, symptom (such as fever, abdominal pain, diarrhea, shock, etc), and laboratory data were collected from the electronic medical record. For the 115 patients and 20 laboratory values, there was a total of 130 missing values (5.7%). Missing laboratory values were imputed using the median value based on the presence or absence of MIS-C. The data were split into a training (93 patients, 80%) and testing (22 patients, 20%) set. The training set was used to train a random forest model and the testing set was used to evaluate model performance. R 4.0.2 was used for modeling with the following packages: tidymodels and randomForest.

**Results:**

There were 115 patients of which 49 were females, and 77 were diagnosed with MIS-C. The median age of the patients with MIS-C was 115 months and 79 months for those without MIS-C. In the testing set, all 15 patients with MIS-C were classified correctly but of the 7 without MIS-C, the model predicted 4 of the patients correctly. This gives a sensitivity of 100% and specificity of 57%. When changing the seed and testing set, the sensitivity remained 100% but the specificity improved to 86%. The random forest algorithm showed that the most important features were pro-calcitonin, ferritin, pro-BNP, and CRP.

**Conclusion:**

During the height of the SARS-CoV-2 pandemic, many children were being admitted with suspected MIS-C, but clinicians struggled to confirm the diagnosis. We have found a model predicting which of these patients had MIS-C with high sensitivity. This model is a first step of many toward creating the foundation of personalized medicine for children.

**Disclosures:**

**All Authors**: No reported disclosures

